# Pulmonary Hypertension in COPD: A Case Study and Review of the Literature

**DOI:** 10.3390/medicina55080432

**Published:** 2019-08-02

**Authors:** Steven J. Cassady, Robert M. Reed

**Affiliations:** Department of Pulmonary & Critical Care Medicine, University of Maryland School of Medicine, Baltimore, MD 21201, USA

**Keywords:** chronic obstructive pulmonary disease, pulmonary hypertension, PH-COPD

## Abstract

Pulmonary hypertension (PH) is a frequently encountered complication of chronic obstructive pulmonary disease (COPD) and is associated with worsened clinical symptoms and prognosis. The prevalence of PH-COPD is not concretely established as classification criteria vary historically, but the presence of severe disease out of proportion to underlying COPD is relatively rare. Right heart catheterization, the gold standard in diagnosis of PH, is infrequently performed in COPD, and the overlap in the clinical symptoms of PH and COPD presents diagnostic challenges. Proven treatments are limited. Trials exploring the use of vasodilator therapy in this patient group generally demonstrate improvements in hemodynamics accompanied by worsening gas exchange without clearly demonstrated improvements in clinically meaningful outcomes. In-depth workup of underlying pulmonary hypertension and use of pulmonary vasodilator medications may be appropriate on an individual basis. We present a case study and a review and discussion of the pertinent literature on this topic.

## 1. Case Study

A man in his 60s with longstanding chronic obstructive pulmonary disease (COPD) presents himself as a new patient. He has a medical history of New York Heart Association (NYHA) Class I heart failure with a preserved ejection fraction of 60%, hypertension, hyperlipidemia, and a cerebrovascular accident six years ago with no residual deficits. He has smoked a pack of cigarettes a day from the age of 22 until quitting smoking at age 60 when he had a stroke. He indicates that he has been well-controlled on maintenance inhaler therapy, including a long-acting beta-agonist, long-acting muscarinic agent, and inhaled corticosteroid, and has never required oxygen therapy. His ability to perform daily activities is nevertheless somewhat limited by exertional dyspnea. His most recent pulmonary function testing indicates an forced expiratory volume in 1 second (FEV_1_) of 1.98 L (63% of predicted) and a forced vital capacity (FVC) of 3.24 L (79% of predicted), both values obtained post-bronchodilator, with mildly elevated total lung capacity and residual volume suggesting hyperinflation and air trapping. The diffusion capacity of carbon monoxide on this testing is markedly reduced at 38% of predicted.

He reports a long period during which exacerbations of his COPD were infrequent, with none requiring hospitalization. However, about six months ago, he began to have gradually worsening exercise tolerance for which his inhalers seem to be largely ineffective and he has required two brief hospitalizations for acute exacerbations that were treated with nebulized bronchodilators, corticosteroids, and antibiotics. Concerned about progression of his congestive heart failure, his cardiologist sent him for a repeat transthoracic echocardiogram, which showed no appreciable change in the systolic or diastolic function of the left ventricle but showed new moderate dilation and moderate systolic dysfunction of the right ventricle. Right ventricular systolic pressure could not be measured due to lack of a tricuspid regurgitant jet. Concerned about his progressive symptoms, he presented himself to the outpatient pulmonary practice, where his resting oxygen saturation today is 91%. He has heard some mention of pulmonary hypertension and would like to discuss that topic as it pertains to him.

While the above case is hypothetical, it is typical of our clinical experience and introduces the theme of this review.

## 2. Discussion and Review of the Literature

### 2.1. Overview, Classification, and Epidemiology

Pulmonary hypertension (PH) has traditionally been defined by a mean pulmonary artery pressure of greater than 25 mmHg, though recent work to further classify the disease by the Sixth World Symposium on Pulmonary Hypertension has suggested a lower threshold of 20 mmHg along with pulmonary vascular resistance (PVR) of ≥3 Wood units for pre-capillary disease [1]. PH exists both as a discrete disease, as in pulmonary arterial hypertension (WHO Group I), or as a disease process attributable to other chronic diseases including chronic cardiac and pulmonary disease.

Efforts to classify pulmonary hypertension in COPD by a 2013 task force proposed to conceptually divide affected patients into two groups: (1) PH-COPD defined in a COPD patient by a mean pulmonary artery pressure (mPAP) ≥ 25 mmHg; and (2) severe PH-COPD, defined by the presence of mPAP ≥ 35 mmHg or ≥ 25 mmHg with the presence of a low cardiac index of <2.0 L/min/m^2^ [2]. The task force suggested that the severe group of patients represented an important minority with disproportionately high rates of vascular remodeling and a loss of circulatory reserve that outpaced the loss of ventilatory reserve. The task force further suggested that this group should be the focus for studies that explore the use of pulmonary vasodilator medications in COPD patients. Indeed, available data suggest that severe PH-COPD represents a phenotype with markedly worsened exercise capacity and prognosis [3].

Prevalence estimates for PH in patients with COPD are not well-established, as right heart catherization is not routinely performed in this patient population and echocardiography is subject to diagnostic limitations. Estimation is further complicated by the use of a variety of different cutoff values to establish the presence of pulmonary hypertension in prior studies. A 1981 study of 175 patients with moderate-to-severe COPD who underwent right heart catheterization found that 35% had a mean pulmonary artery pressure of >20 mmHg [4]. At the higher end of estimation, a study of 120 patients with severe emphysema with an average FEV1 of 27% being evaluated for lung volume reduction surgery (LVRS) found that 90.8% of patients had mPAP > 20 mmHg [5]. Notably, no correlation was found between severity of emphysema and pulmonary artery pressure in this patient group. A study of 998 COPD inpatients admitted for respiratory failure with a mean FEV1 of 33% demonstrated that, while the mean mPAP was 20.3 mmHg, only 2.7% had severe PH, defined as an mPAP ≥ 40 mmHg, with only 1.1% of these patients having only COPD as an attributable cause of PH [6]. Overall, while elevated mean pulmonary artery pressures appear to be reasonably common in COPD, severe PH out of proportion to underlying lung disease is relatively rare.

### 2.2. Pathophysiology

In PH secondary to COPD, ongoing chronic hypoxic pulmonary vasoconstriction leads to changes that produce fixed remodeling of the pulmonary vasculature, namely fibromuscular intimal thickening and an increase in smooth muscle of the media of the pulmonary arterioles and arteries [7]. These same changes have also been noted in the pulmonary vasculature of smokers even without the presence of airflow obstruction [8]. Increased muscular hyperplasia of the microvasculature and decreases in alveolar capillary density have been found in severe PH-COPD patients, possibly representing a specific subgroup with regard to severity [9]. Loss of pulmonary vessels, long proposed as an underlying pathological feature of emphysema, may also occur and has been suggested as the key pathophysiological feature of a subtype of PH strongly associated with smoking and low diffusion capacity [10,11]. Increased levels of pulmonary vasoconstrictive mediators including endothelin-1 and decreased expression of endothelial nitric oxide synthase and prostacyclin synthase have also been observed in COPD compared to normal patients [12,13,14,15]. Genetic determinants also appear to play a role in the differential development of PH in patients with chronic lung disease [16].

The natural history of PH in COPD may begin with exercise-induced PH that precedes PH at rest. A study of 131 patients with COPD showed that in patients with mild-to-moderate COPD, progression of increases in right-sided pressures was slow, at roughly 0.4 mmHg per year, and that only about 25% of COPD patients with mild-to-moderate hypoxemia were found to have developed resting PH by six years. However, the presence of exercise-induced PH conferred a substantially greater risk of eventually developing resting PH [17].

These changes as a whole result in increased pulmonary vascular resistance and increased demand on the right ventricle. With progression of disease, the right ventricle may become overtly decompensated, resulting in cor pulmonale.

### 2.3. Diagnosis

Differentiating symptoms such as dyspnea on exertion or chest tightness as PH from underlying advanced COPD is challenging. The typical physical exam findings of precapillary pulmonary hypertension, such as a loud P2 and/or a holosystolic murmur of tricuspid regurgitation, may be less prominent in COPD patients or more difficult to appreciate due to distant heart sounds on exam. In addition, right heart pressures may not be elevated enough to produce these physical exam findings except during acute exacerbations when pulmonary pressures are demonstrably higher.

Spirometry values have not been shown to reliably correlate with the presence of underlying PH. Significant decrements in diffusion capacity for carbon monoxide (DLCO) may be suggestive of PH, though this is non-specific as this can also be observed in severe emphysema. However, the presence of a disproportionately reduced DLCO with respect to spirometric and radiographic changes of emphysema may be an indication of PH out-of-proportion to underlying COPD that would merit further investigation and treatment [6].

As discussed above, right heart catheterization, the gold standard for diagnosis of PH, is infrequently performed in patients with COPD unless specific cardiac indications exist, and there are currently no studies demonstrating its clinical usefulness in the routine evaluation of COPD.

Echocardiography represents a useful but limited modality to diagnose PH in the COPD population, with estimation of right ventricular systolic pressure (RSVP) from the velocity of tricuspid regurgitation considered to be a reliable method of detection when this signal is present [18]. However, in a study of 192 patients with advanced lung disease, including severe COPD, and 50 healthy controls, it was found that tricuspid regurgitation, which is critical for ultrasonographic assessment of PH, could not be assessed in 52% of patients [19]. In addition, parameters of right heart enlargement and dysfunction alone lacked sufficient specificity to reliably indicate PH in this patient population. Finally, the presence of gas trapping or hyperinflation may limit the echocardiographic technique and prevent accurate estimation of these values, with specificity for detection of PH of only 55% reported in one study of 374 patients with advanced lung disease, of whom over half (68%) had obstructive lung disease [20].

Imaging modalities such as computed tomography (CT) may offer promise for noninvasive diagnosis of pulmonary hypertension. A study of 60 patients with severe COPD (FEV1 of 27% ± 12%) found a linear correlation between the ratio of pulmonary artery to ascending aorta diameter with mean PA pressure, while such a correlation could not be observed using pulmonary artery systolic pressure (PASP) derived from echocardiography. However, the described area under the curve (AUC) of 0.83 is likely insufficient for truly accurate screening [21].

### 2.4. Treatment

Treatment options for COPD-PH remain limited outside of the routine inhaled medications to treat underlying COPD. Long-term oxygen therapy (LTOT) improves survival in COPD patients with hypoxemia and has also been shown to reduce mPAP over a six-month period, presumably by attenuating hypoxic pulmonary vasoconstriction [22]. However, LTOT does not have proven survival benefits in those with baseline oxygen saturations above the threshold of 89% [23].

Formal studies of vasodilator therapy in PH-COPD have been largely disappointing, with many demonstrating a measurable improvement in hemodynamics accompanied by worsening hypoxemia due to altered ventilation-perfusion matching, though some trials have shown no net change in oxygenation. Improvements in hemodynamics in these groups do not appear to consistently confer an improvement in symptoms, and some studies have noted worsening exercise tolerance with their use. Trials using pulmonary vasodilators in PH-COPD have also generally been limited by a short trial duration and small numbers of patients, limiting the strength of their conclusions.

Phosphodiesterase-5 inhibitors are often used as first-line therapy in pulmonary arterial hypertension and have been explored by a number of trials in PH-COPD patients. The Sildenafil and Pulmonary HypERtension in COPD (SPHERIC-1) trial was a double-blind, randomized, placebo-controlled trial of 28 patients with PH-COPD (FEV1 of 54% ± 22% in the sildenafil group) that followed patients for 16 weeks on sildenafil 20 mg three times daily [24]. Significant improvements in hemodynamics, including decreases in mPAP and pulmonary vascular resistance (PVR) and increases in cardiac index, were noted, and no detrimental effects on gas exchange or hypoxemia were found. In addition, BODE index and mMRC score improved, the latter by −0.51 in the experimental group. Notably, this study group had less severe baseline COPD than those typically studied in pulmonary vasodilator trials, with patients with FEV1 < 30% excluded, suggesting that ventilation-perfusion alterations may be subtler and better tolerated in less severe disease. A double-blind and placebo-controlled randomized controlled trial of 10 mg of daily tadalafil in 120 patients with COPD (mean FEV1 of 40%) and PH (measured by RVSP > 30 mmHg on echocardiography) described small improvements in right heart hemodynamics on echo but no improvement in exercise capacity or quality of life. There were no significant differences in SpO2 at the end of the 12-week study [25].

Endothelin receptor antagonists (ERAs) have also been studied with similar equivocal results. A study by Stolz et al. evaluated the use of bosentan, an endothelin receptor antagonist, in a double-blind randomized trial of 30 patients with severe or very severe COPD [26]. Of the 20 patients assigned to the bosentan group, six stopped the medication due to adverse side effects prior to the end of the trial. Six-minute walk time actually decreased in the bosentan group, as did mean PaO_2_. Notably, this study was limited by its lack of formal evaluation of the included patients for underlying PH prior to its initiation. A later randomized though not double-blinded trial of 32 COPD patients with confirmed PH (mPAP ≥ 25 mmHg on right heart catheterization) noted improvements in mPAP and PVR in the bosentan group as well as a slight improvement in BODE scores and did not show significant differences in PaO_2_ at 18 months, suggesting greater promise for ERAs in a more select group of PH-COPD patients [27].

Inhaled pulmonary vasodilators have also been studied to a more limited degree in PH-COPD patients. Wang et al. studied the efficacy and safety of inhaled iloprost, a synthetic prostacyclin, in 67 PH-COPD patients, 37 of whom had severe disease by the above criteria and found an average decrease in mPAP of −2.1 mmHg and increase in cardiac output of 0.4 L/min after a single 20 µg nebulized dose without significant changes in PaO_2_ or PaCO_2_ [28]. A 2016 study of inhaled treprostinil in a small group of nine PH-COPD patients also found no change in oxygenation based on arterial blood gas but unexpectedly showed decreases in FEV1, FVC, and DLCO [29].

Real-world use of pulmonary vasodilator medications in PH-COPD has also met with mixed results. Of the 101 patients included in the ASPIRE registry, 43 patients with severe PH-COPD were treated compassionately, mostly with PDE-5 inhibitors, for a duration of at least three months. Survival among these patients was only 72% at one year, not significantly different from the 16 untreated patients despite the treatment group having significantly worse baseline hemodynamics. Of these 43 patients, eight of them showed evident clinical improvement and did show an improvement in survival, suggesting as yet undetermined factors in their disease that rendered vasodilator therapy more beneficial [3]. Of particular interest, the study did not note significant perturbations in oxygen saturation in patients on vasodilator therapy, though the authors caution that the study was not designed to assess this.

### 2.5. Prognosis

The negative prognostic implications of an elevated mean pulmonary artery pressure in COPD are well-understood. A 1981 study by Weitzenblum et al. of 175 patients with moderate-to-severe COPD found that survival rates were markedly lower in those that had mPAP above 20 mmHg at four and seven year follow-up, with survival rates at four years of 71.8% in those with mPAP < 20 mmHg and 49.4% in those above 20 mmHg [4]. In this study, mPAP was as strong a factor for predicting survival as PaO_2_, PaCO_2_, and FEV1. More recently, the ASPIRE registry found a 3-year survival of only 33% in those with severe PH-COPD, defined as mPAP of 40 mmHg or greater [3].

Many recent studies have suggested that the burden of mortality in PH-COPD is at least as high if not higher than that of idiopathic PAH (IPAH), itself a disease with notoriously high mortality rates. A 2015 study of 1472 PH patients included in the COMPERA registry revealed a 3-year survival rate of 70.7% in IPAH but only 58.8% in PH-COPD [30]. Rates of mortality were noted to be lower at one, two, and three years in PH-COPD compared to PH-ILD. A 2019 study comparing 51 patients with PH due to chronic lung disease to 83 patients with IPAH demonstrated that the former patient group had equally poor outcomes despite lower mean pulmonary artery pressure and pulmonary vascular resistance [31]. A PVR of seven Wood units or greater was noted to confer a 3-fold higher risk of mortality and was more strongly associated with mortality than mPAP. Similar to the COMPERA registry above, 5-year survival was significantly lower amongst those with PH-ILD compared to PH-COPD.

In addition to markedly worsened overall survival, the presence of pulmonary artery enlargement on CT, implicative of higher mean pulmonary artery pressure, is associated with increased risk of severe COPD exacerbations requiring hospitalization [32].

## 3. Return to the Case Study

Given the presence of hypoxemia and severe dyspnea out of proportion to the spirometric defects present in the patient, as well as the severe defect in diffusion capacity, the possibility of significant pulmonary hypertension secondary to COPD is felt to be a reasonable diagnostic possibility. A repeat non-contrast CT scan of the chest that is obtained to evaluate for concurrent interstitial lung disease demonstrates emphysematous changes more prominent in the upper lobes; enlargement of the main pulmonary artery is also noted compared to on the last CT scan performed roughly one year prior for lung cancer screening.

A discussion between the patient, pulmonologist, and cardiologist is held regarding the risks and benefits of right heart catheterization, given that echocardiography suggested but could not estimate right heart pressures and a repeat CT scan demonstrated an interval increase in the width of the main pulmonary artery.

The patient consents to undergo right heart catheterization, which reveals a mean pulmonary artery pressure of 37 mmHg and a pulmonary capillary wedge pressure of 13 mmHg. Vasoreactivity testing was not performed. Alternative etiologies to explain this underlying pulmonary hypertension are discussed. The patient reveals he underwent polysomnography roughly two and a half years prior that did not demonstrate obstructive sleep apnea. A repeat STOP-BANG screening in clinic results in a score of 2, and the decision is made not to repeat sleep testing. A ventilation-perfusion scan to rule out chronic thromboembolic pulmonary disease (CTEPH) is considered, but the patient denies any history of thromboembolic disease.

Having effectively ruled out other likely contributing factors, the elevated right heart pressures are attributed to the known underlying lung disease and the diagnosis of severe PH-COPD is made. A trial of sildenafil (20 mg) three times daily is begun; after four weeks, the patient reports a mild improvement in exertional dyspnea but is noted to have a slight worsening in baseline oxygen saturation to 89%. Despite this change, he opts to continue the medication in light of his perception of a slight increase in exercise tolerance. In light of this worsening in baseline oxygen saturation, he is started on 2 L/min supplemental oxygen. He is offered referral to pulmonary rehabilitation but declines.

After six months, he returns to clinic and reports that both his ability to perform activities of daily living and his exertional dyspnea have not changed, though the purchase of a portable oxygen concentrator has made leaving his home easier. His oxygen saturation is 93% on 2 L/min in the office. A repeat echocardiogram is ordered that demonstrates continued moderate right ventricular systolic dysfunction, but mild dilatation of the ventricle compared to the moderate dilatation shown in the previous study; a tricuspid regurgitant jet again cannot be measured. A repeat six-minute walk distance is shown to be 37% of predicted from the 34% six months prior. In light of the patient’s lack of significant clinical improvement and his concerns of the cost of ongoing sildenafil therapy, the medication is stopped, and he is continued on his inhaled regimen for COPD and supplemental oxygen.
